# Supplementary material for: Visually constructing the chemical structure of a single molecule by scanning Raman picoscopy
Source: Natl Sci Rev. 2019 Nov 8;6(6):1169–75. doi: 10.1093/nsr/nwz180 (PMC8291412; doi:10.1093/nsr/nwz180)
Supplement: nwz180_Supplemental_File [file nwz180_supplemental_file.docx]

Supplementary Materials for

Visually Constructing the Chemical Structure of a Single Molecule by Scanning Raman Picoscopy

Yao Zhang^†^, Ben Yang^†^, Atif Ghafoor^†^, Yang Zhang, Yu-Fan Zhang, Rui-Pu Wang, Jin-Long Yang, Yi Luo^*^, Zhen-Chao Dong^*^ and J. G. Hou^*^

Hefei National Laboratory for Physical Sciences at the Microscale and Synergetic Innovation Center of Quantum Information and Quantum Physics, University of Science and Technology of China, Hefei, Anhui 230026, China.

^*^Corresponding authors: E-Mails: [yiluo@ustc.edu.cn](mailto:yiluo@ustc.edu.cn); [zcdong@ustc.edu.cn](mailto:zcdong@ustc.edu.cn); [jghou@ustc.edu.cn](mailto:jghou@ustc.edu.cn)

^†^Contributed equally to this work.

S1. Experimental setup

Our STM-controlled SRP experiments were performed on a custom-built optical-STM system operating under ultrahigh vacuum (~5.0×10^−11^ Torr) and at liquid-helium cryogenic conditions (~7 K), as schematically shown in Fig. S1. The thermal drift is about 50 pm/h at ~7 K under laser illumination, which is almost negligible for the measurement time used in the present work. MgP molecules were thermally evaporated onto the Ag(100) surface at about 7 K, which was previously cleaned by cycles of argon ion sputtering and annealing. Electrochemically etched sliver (Ag) tips were used in all our experiments. The tip apex was cleaned by electron-bombarding and argon-ion sputtering inside UHV, followed by further atomistic modification through tip indentation to achieve SRP-active status for efficient light coupling. The last step to tune the tip status by tip indentation, likely producing an atomistic protrusion at the very end of the tip apex, typically takes about 10 min for a good tunable tip. Once a SRP-active tip is obtained in this way, we can routinely carry out SRP imaging with a spatial resolution at the Ångstrom level. We would like to highlight two advantages in using lower-temperature conditions at liquid helium (~7 K): (1) reducing the thermal drift and thus improving the stability of the STM junction; (2) suppressing both the molecular diffusion on the surface and the motions of constituent atoms and groups within a molecule and thus improving the stability of the molecule and local molecular orientations under study. Such greatly improved stability of the whole system enables us to approach the tip closer to the molecule (e.g., ~2.9 Å for the tip‒substrate distance at the experimental condition of −0.02 V and 8 nA), leading to better confined local electric field and higher spatial resolution in addition to greatly improved spectral signal-to-noise ratios.

A single-longitude-mode diode-pumped laser at 532 nm (CrystaLaser, CL532−100-SO) was used to provide a linearly polarized laser beam (>300:1) for Raman excitation. The beam was fiber-coupled to a dark-box via a single-mode polarization-maintaining fiber with a collimated output beam of ~1.0 mm in diameter. A round continuously variable metallic neutral density filter was used to adjust the laser output power. Two reflective mirrors were used to provide freedoms for optical alignment. A half-wave plate was used to achieve desired *p*-polarization for incident laser. The laser beam was introduced into the UHV chamber via a quartz viewport after reflected by a 30:70 (R:T) beam splitter. Another beam splitter, which was removed during Raman measurements, was positioned in the optical collection path to help monitor the focusing of the laser beam into the tunnel junction with a video camera. The collimated beam was re-focused by an aspheric lens (*f*=12.4 mm, NA=0.46) into the tip−substrate junction with an angle of 60° from the surface normal. The diameter of the laser spot on the sample surface was about 30 μm. The Raman scattered light was collected by the same lens, transmitted through the 30:70 (R:T) beam splitter, filtered by an edge filter to remove residual laser light, and finally fiber-coupled to a spectrometer using a slit size of 100 μm. The Raman signal light was dispersed by a 600 grooves/mm grating and detected by a liquid-nitrogen cooled charge-coupled-device (CCD) (Princeton Instrument). The laser power used in our SRP experiments was about 0.5 mW. The spectral resolution was about 18 cm^−1^, limited by the instrumental resolution as a result of the trade-off between the photon collection efficiency and the detection of Raman photons with a reasonable signal-to-noise ratio. The STM operation was controlled by Nanonis-SPECS electronics. The Raman spectral mapping, namely the SRP imaging, was carried out through a synchronization function between the STM controller and CCD camera, acquiring a spectrum at each pixel during scanning [1]. All STM imaging and Raman spectral measurements presented here were carried out in a constant-current mode with the sample biased. All Raman spectra presented here were not corrected for the wavelength-dependent sensitivity of photon detection systems.

**
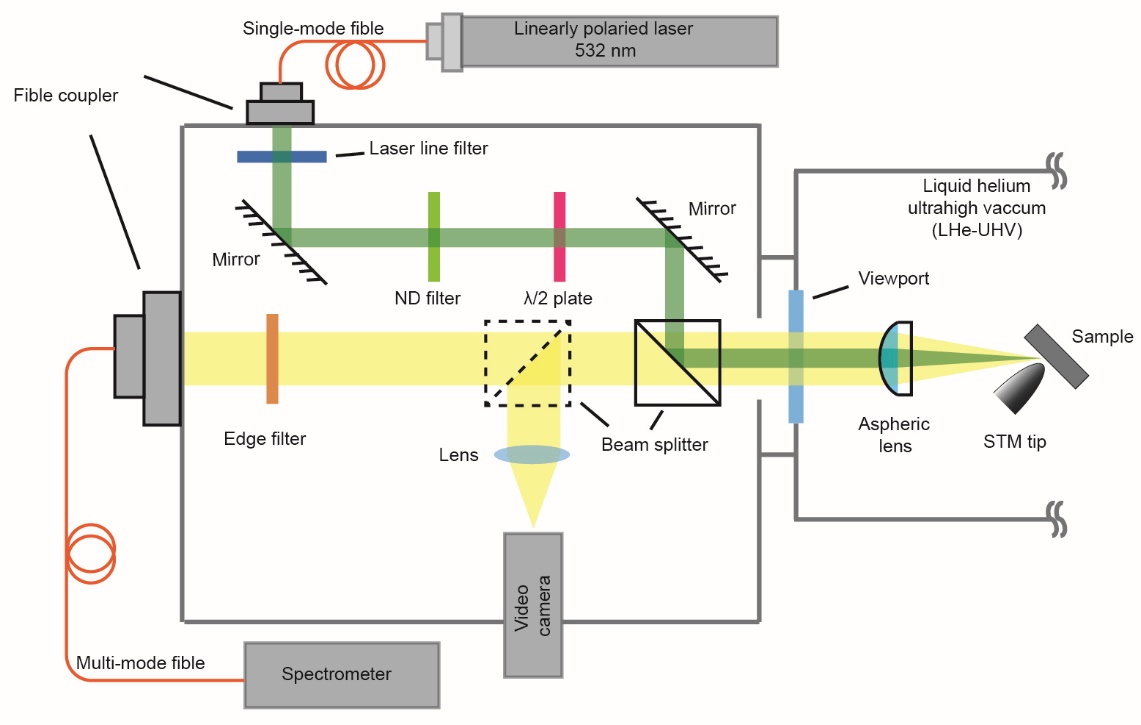
**

Figure S1. Schematic drawing of our custom-built experimental setup for SRP measurements. The setup is composed of four sub-systems: a laser source for Raman excitation, a dark-box for optical filtering and alignment, a low-temperature (LT) ultrahigh-vacuum (UHV) scanning tunneling microscope (STM) for sample preparation and characterization with a built-in lens for both Raman excitation and collection, and a spectrometer equipped with a highly sensitive CCD detector for Raman spectral measurements.

**S2. Interference effect in Raman scattering from a single molecule within a local electric field**

It should not be additional discussion, analysis, interpretation, or critique. Based on the model proposed by Duan *et al*. [2, 3], the polarizability for Raman scattering in a local electric field can be expressed as

$\frac{\partial\alpha_{pq}^{\text{loc}}}{\partial Q_{k}}=\sum_{r} \frac{2}{\Delta E_{rg}}\left(\frac{\partial\langle\psi_{g}|\hat{p}|\psi_{r}\rangle}{\partial Q_{k}}\langle\psi_{r}|\hat{q}g\left(\mathbf{r}-\mathbf{R}_{0}\right)|\psi_{g}\rangle+\langle\psi_{g}|\hat{p}|\psi_{r}\rangle\frac{\partial\langle\psi_{r}|\hat{q}g\left(\mathbf{r}-\mathbf{R}_{0}\right)|\psi_{g}\rangle}{\partial Q_{k}}\right)$, (S1)

where $\Delta E_{rg}$ is the vertical excitation energy between the ground states $|\psi_{g}\rangle$ and intermediate state $|\psi_{r}\rangle$, *p* and *q* are Cartesian coordinates, *g*(**r**−**R**_0_) is the spatial distribution of the local electric field amplitude centered at the tip position **R**_0_, and *Q_k_* corresponds to the *k*-th vibrational normal mode. In the representation of atomic orbital basis, the integral term can be expressed as

, (S2)

, (S3)

where *C_α_*(*_β_*) is the coefficient for corresponding electronic states and *φ_χ_*(*_ν_*) is the atomic orbital. Considering the highly confined nature of the local plasmonic field (with the direction along the *z*-axis), we can approximate the field distribution function *g*(**r**) as $g\left(\mathbf{r}\right)=\delta\left(\mathbf{r}-\mathbf{R}_{0}\right)$ where **R**_0_ is the center of the local field defined by the position of tip. Then the integral in Eq. (S3) can be simplified as

for *α*=*β*: , (S4a)

for *α*≠*β*: . (S4b)

In this sense, the polarization of the single molecule would be non-zero only when the atomic wavefunction () or the overlap integral () has values at the position beneath the tip. In other words, the atomistic tip would only resolve single atoms or the chemical bonds between the atoms. The local electric field in reality cannot behave as a *δ*-function but has a spatial extent, so the contributions from nearby atoms and chemical bonds in the proximity should be included as well. By defining Δ**R**_0_ as the spread width of the local field, Eq. (S4) can be rewritten as

, (S5)

in which the overlap integral contributions of atomic wavefunctions within the region **R**_0_+Δ**R**_0_ are all considered. Therefore, the effective polarizability can be finally expressed as the sum-over contributions from all atoms and overlaps as

$\frac{\partial\alpha^{\text{loc}}\left(\mathbf{R}_{0}\right)}{\partial Q_{k}}=\sum_{\alpha} \frac{\partial\alpha_{\alpha\alpha}^{\text{loc}}\left(\mathbf{R}_{0}+\Delta\mathbf{R}_{0}\right)}{\partial Q_{k}}+\sum_{\alpha} \sum_{\beta\neq\alpha} \frac{\partial\alpha_{\alpha\beta}^{\text{loc}}\left(\mathbf{R}_{0}+\Delta\mathbf{R}_{0}\right)}{\partial Q_{k}}$. (S6)

Here the first term can be regarded as the contributions from single atoms, and the second term is the contribution from the overlap between atoms *α* and *β*.

The influence of vibrations from atomic contributions can be considered by expanding the derivative of $\alpha_{\alpha\beta}^{\text{loc}}$ in atomic coordinates as [4, 5]

$\frac{\partial\alpha_{\alpha\beta}^{\text{loc}}}{\partial Q_{k}}=\sum_{\alpha'} \sum_{i=x,y,z} \frac{\xi_{k}^{\alpha',i}}{\sqrt{\mu_{k}}}\left(\frac{\partial\alpha_{\alpha\beta}^{\text{loc}}}{\partial\chi_{\alpha',i}}\right)$, (S7)

where $\xi_{k}^{\alpha',i}$ is the displacement of atom *α'* along the *i*-axis corresponding to the *k*-th vibrational mode, *μ_k_* is the reduced mass corresponding to the *k*-th vibrational mode, and $\chi_{\alpha',i}$ is the coordinate along the *i*-axis of atom *α'*. Within this framework, only the contributions from atoms within the local field region need to be considered for the evaluation of $\alpha_{\alpha\beta}^{\text{loc}}$. In other words, *only the atomic vibrations within the local field region would contribute dominantly to the Raman signals from a single molecule*.

If the local field distribution is not a well-confined *δ*-function but has certain spatial extent, the overall Raman signals would be contributed by a sum-up of neighboring atoms and chemical bonds. Considering different phases and directions of atomic displacements corresponding to different vibration modes (namely associated with symmetric and anti-symmetric vibrations), an interference effect is expected to occur, which can be understood as follows. As an example, let us consider the two C−H bonds (named as C_1_−H_1_ and C_2_−H_2_) corresponding to the C−H stretching vibration within one pyrrole ring that is shown in Fig. 2 of the main text. If these two C−H bonds vibrate independently without any interactions, their vibrational frequencies should be completely identical. The mutual interaction between their vibrations will result in a splitting of the frequency: one high-energy mode corresponding to the symmetric vibration of two C−H bonds with in-phase stretching, and one low-energy mode corresponding to the anti-symmetric vibration of two C−H bonds with out-of-phase stretching. Following Eq. (S7), we can express the above process in C−H bond coordinates as

$\frac{\partial\alpha^{\text{loc}}}{\partial Q_{k}}=\frac{1}{\sqrt{\mu_{k}}}\left[\delta d_{\text{C}_{1}-\text{H}_{1}}\left(\frac{\partial\alpha^{\text{loc}}}{\partial\chi_{\text{C}_{1}-\text{H}_{1}}}\right)+\delta d_{\text{C}_{2}-\text{H}_{2}}\left(\frac{\partial\alpha^{\text{loc}}}{\partial\chi_{\text{C}_{2}-\text{H}_{2}}}\right)\right]$, (S8)

where $\delta d_{\text{C}-\text{H}}$ is the change of the C−H bond length ($\delta d_{\text{C}_{1}-\text{H}_{1}}=\delta d_{\text{C}_{2}-\text{H}_{2}}$for symmetric vibration and $\delta d_{\text{C}_{1}-\text{H}_{1}}=-\delta d_{\text{C}_{2}-\text{H}_{2}}$ for anti-symmetric vibration). If the tip is located above one single C−H bond, only the first (or second) term dominates, indicating the observation of a single-bond vibration. However, if the tip is located in the middle between two C−H bonds, the resultant derivative value $\partial\alpha^{\text{loc}}/\partial\chi_{\text{C}-\text{H}}$ would be similar for both bonds and the above equation can result in two different situations for symmetric and anti-symmetric vibrations as

symmetric: $\frac{\partial\alpha^{\text{loc}}}{\partial Q_{k}}=\frac{2}{\sqrt{\mu_{k}}}\delta d_{\text{C}-\text{H}}\left(\frac{\partial\alpha^{\text{loc}}}{\partial\chi_{\text{C}-\text{H}}}\right)$, (S9)

anti-symmetric: $\frac{\partial\alpha^{\text{loc}}}{\partial Q_{k}}=0$. (S10)

For the symmetric mode the contributions from two stretching C−H bonds will sum up and result in a constructive interference phenomenon, while for the anti-symmetric mode the contributions from two stretching C−H bonds will cancel out each other, resulting in a destructive interference phenomenon. In other words, the in-phase local vibrations carry the same sign in polarization, while the out-of-phase local vibrations carry the opposite signs, giving the null integral value under the sampling window of the local field. Such interference effect also occurs for other vibrational modes, thus providing a theoretical basis to explain the various patterns in the observed SRP mapping images.

**S3. More details on the vibrations of typical Raman modes**

In the main text, we have used only four typical types of SRP images to construct the chemical structure of the target molecule. In fact, all the other SRP images coincide with the determined Mg-porphine structure. For example, in Fig. 3 in the main text, we have discussed the pyrrole ring vibrations and made assignments for four typical Raman peaks at 1359 cm^−1^, 1377 cm^−1^, 1463 cm^−1^ and 1499 cm^−1^ based on the combination of symmetric and anti-symmetric relations for four pyrrole-ring vibrations. We notice that there is another pronounced Raman peak at 1475 cm^−1^ in this region. The observed SRP image for this mode also exhibits a “four-lobe” feature, suggesting its correlation with the vibrations of the pyrrole rings, but probably with different phase relations. Judging from both the absence of Raman intensity at the center and noticeable intensities at the gap positions, this mode is likely to be a combination of two orthogonal vibration modes, as shown in Fig. S2. Because of the two-fold degeneracy, the obtained SRP image is in fact an overlay of two orthogonal patterns. [We would like to make some notes on the selection rule issue in the SRP setup. When an atomically sharp tip is brought very close to a molecule, the spatial distribution of the local electric field is highly confined to a region that could be even smaller than the molecular size. In this context, the contributions to the polarizability from different parts of the whole molecule would not be added or cancelled because the field distribution is not uniform anymore, which could change the selection rules to determine whether a vibration mode is Raman active or not. In addition, the contribution of the field-gradient term to the polarizability may also become important for the highly confined field.]


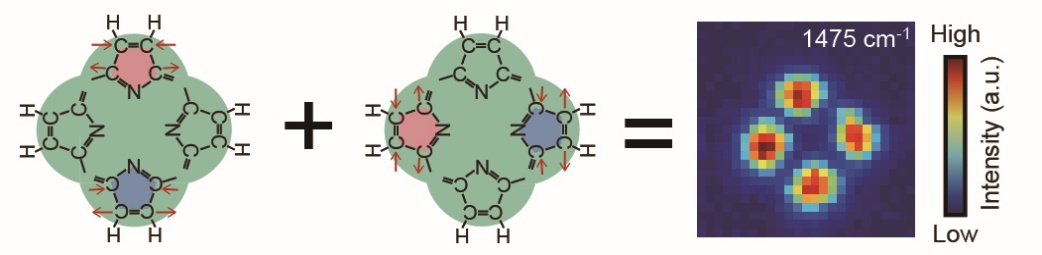


Figure S2. Vibration analysis for the Raman mode at 1475 cm^−1^. The SRP image on the right exhibits a “four-lobe” feature, which can be assigned to two-fold degenerated vibrations, as shown on the left. The pink (blue) pentagon represents the dominant “compression” (“stretching”) motion of the pyrrole ring, showing out-of-phase relations for the vibrations of opposite pyrrole rings labelled (the unlabeled pyrrole rings do not vibrate in this mode).

In the following, we provide more detailed schematics illustrating the vibrational modes mentioned in Fig. 4 of the main text for the bridging units and central metal atom. The out-of-plane bending vibrations related to the bridging C−H bonds are illustrated in Fig. S3. For the SRP image at 841 cm^−1^, the main out-of-plane vibration is contributed by the bridging C−H bonds (Fig. S3A). For the Raman peak at 925 cm^−1^, the SRP mapping image indicates that the out-of-plane vibration of the bridging C−H bonds is accompanied by the in-phase out-of-plane vibration of adjacent C−H bonds in the pyrrole rings (Fig. S3B). On the other hand, for the low-wavenumber region associated with the vibrations of the central metal atom, the low-energy Raman peaks at 211 cm^−1^ is related to the out-of-plane vibration of the central Mg atom, while the relatively high-energy Raman peak at 361 cm^−1^ is likely to associate with the in-plane vibration of the central atom, as illustrated in Figs. S3C and 3D, respectively.


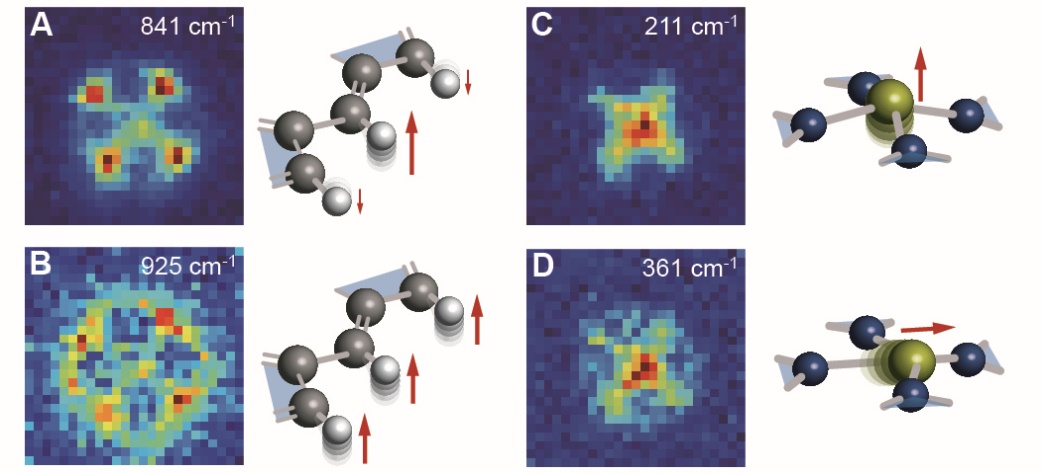


Figure S3. Vibration analysis for the bridging C−H bonds and central metal atom. (A−D) Left: The SRP mapping images for the Raman peaks at 841 cm^−1^, 925 cm^−1^, 211 cm^−1^, and 361 cm^−1^, respectively. Right: The schematics of related vibrations highlighting the motions of related atoms by red arrows. The pyrrole ring planes are denoted by blue shadows in the schematics.

We would to note that the Raman intensity distributions in the mode plots in Fig. 1c are slightly asymmetric, deviating from the ideal four-fold symmetry of a free-space MgP molecule. There are two possible reasons to cause such asymmetries and distortions: One is the adsorption configuration of the molecule. Although the molecule looks very symmetric from the STM topograph, there might be some minor differences in adsorption among different lobes, resulting in some asymmetric features in obtained SRP images. The other reason, which is probably more likely, may be related to the morphology of tip apex. The possible asymmetric structure of the tip apex will modulate the symmetry of the local field and thus affects the excitation and emission processes differently even for symmetric molecular positions, leading to some asymmetries in the SRP images measured.

**S4. Numerical simulation of SRP images for representative Raman modes**

The numerical simulation of the SRP images for different vibrational modes follows the same procedure as in Eq. (S7). The derivative of the polarizability in normal coordinates *Q_k_* can be expanded in atomic coordinates as

$\frac{\partial\alpha}{\partial Q_{k}}=-\sum_{\alpha'} \sum_{i=x,y,z} \frac{\xi_{k}^{\alpha',i}}{\sqrt{\mu_{k}}}\left(\frac{\partial\alpha}{\partial\chi_{\alpha',i}}\right)$, (S11)

in which the derivative term on the right is the value of derivative of polarizability which can be obtained by density functional theory (DFT) calculations. Therefore, the overall polarization process of a single molecule can be described by the polarized dipole moment as

$\mu_{k}=\sum_{\alpha'} \sum_{i=x,y,z} \frac{\xi_{k}^{\alpha',i}}{\sqrt{\mu_{k}}}\left(\frac{\partial\alpha}{\partial\chi_{\alpha',i}}\right)\mathbf{E}_{\alpha'}\equiv\sum_{\alpha'} \mu_{k,\alpha'}$, (S12)

where $\mathbf{E}_{\alpha'}$ is the local electric field at the position of atom *α'*, and $\mu_{k,\alpha'}$ is defined as the polarized atomic dipole moment, which is obtained from the numerical simulation rather than the *δ*-function conceptually assumed above. These atomic dipoles radiate at different frequencies corresponding to different vibrational modes, and the emission is further enhanced by the plasmonic nanocavity, resulting in enhanced Raman signals.

In Fig. S4, we present the comparison between theoretical simulations and experimental SRP images for typical Raman modes. During the simulation by boundary element method [6], an atomistic protrusion with the size of 0.2 nm is introduced at the apex of a tip with a radius of 1 nm and a height of 5 nm. The gap distance between the tip and substrate is set to 0.5 nm, with the molecular plane 0.3 nm above the substrate. The permittivity of Ag is used for both the tip and substrate. The structure of the MgP molecule is optimized in free space and then used to calculate the vibrational properties by DFT with B3LYP/6-311++G(d) basis set in Gaussian 09 [7]. The schematics of five typical vibration modes are illustrated in Fig. S4C (also in Supplementary Video). The simulated SRP images for these modes are plotted in Fig. S4B, showing an excellent agreement with the experimental SRP images observed, which further confirms the experimental observation of full vibrational images and justifies the validity of the methodology proposed in the present work. On the other hand, such an agreement in turn suggests the importance of an atomistic-sharp tip and a very close gap distance in achieving high-resolution imaging down to single-chemical-bond level.


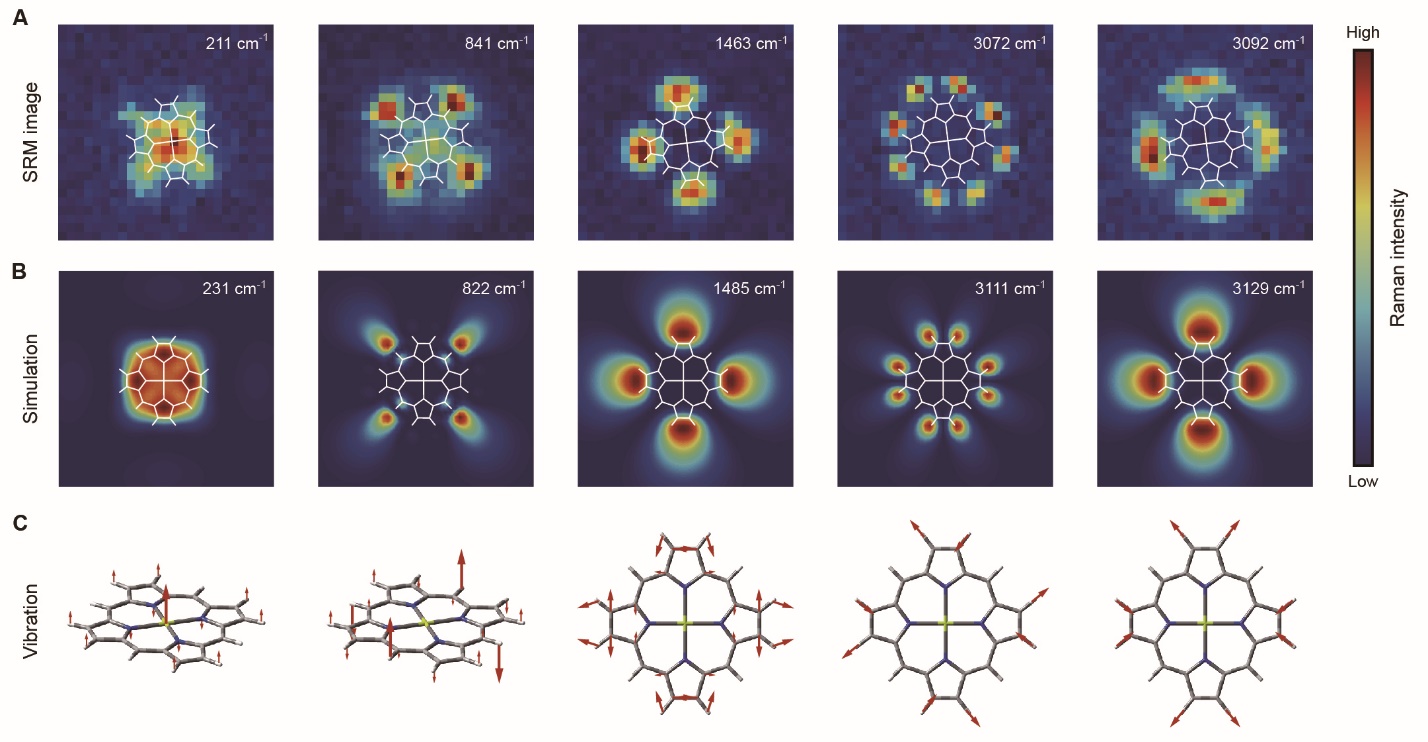


Figure S4. Numerical simulation of SRP images for representative Raman modes. (A) Experimental SRP images at 211 cm^−1^, 841 cm^−1^, 1463 cm^−1^, 3072 cm^−1^ and 3092 cm^−1^. The molecular structure is also overlaid on the simulated and experimental images for comparison. (B) Simulated Raman mapping images by scanning the “atomistic” tip over the single molecule. (C) Schematics of related vibrations highlighting the motions of related atoms by red arrows.

We would like to note that, while SRP image simulations are quite successful, it is still a challenge in the community to simulate site-specific Raman spectra of a single molecule that can agree well with the experimental spectra. This is because the relative intensities of different Raman peaks are determined not only by the intrinsic properties of related vibrational modes, but also influenced by the interaction with substrate as well as technical parameters such as the shape of tip which is usually unknown. In this sense, our high-resolution SRP data provides a test platform for the development of more sophisticated theories.

References

1. Zhang C, Chen LG, Zhang R and Dong ZC. Scanning tunneling microscope based nanoscale optical imaging of molecules on surfaces. *Jpn J Appl Phys* 2015; **54:** 08LA01.
2. Duan S, Tian GJ and Ji YF *et al*. Theoretical modeling of plasmon-enhanced raman images of a single molecule with subnanometer resolution. *J Am Chem Soc* 2015; **137**: 9515–9518.
3. Duan S, Tian GJ and Luo Y. Theory for modeling of high resolution resonant and nonresonant Raman images. *J Chem Theory Comput* 2016; **12**: 4986–4995.
4. Le Ru EC and Etchegoin PG. Principles of surface-enhanced Raman spectroscopy. (Elsevier, Amsterdam, 2009).
5. Decius JC. Compliance matrix and molecular vibrations. *J Chem Phys* 1963; 38: 241–248.
6. Hohenester U and Trügler A. MNPBEM - A Matlab toolbox for the simulation of plasmonic nanoparticles. *Comput Phys Commun* 2012; **183**: 370–381.
7. Frisch MJ *et al.* Gaussian 09 Revision A.02. (Gaussian, Inc., Wallingford, 2009).
